# Artificial Intelligence Tools for Supporting Histopathologic and Molecular Characterization of Gynecological Cancers: A Review

**DOI:** 10.3390/jcm14217465

**Published:** 2025-10-22

**Authors:** Aleksandra Asaturova, João Pinto, António Polonia, Evgeny Karpulevich, Xavier Mattias-Guiu, Catarina Eloy

**Affiliations:** 1 1st Pathology Department, FSBI “National Medical Research Centre for Obstetrics, Gynecology and Perinatology Named After Academician V.I.Kulakov”, The Ministry of Health of the Russian Federation, Akademika Oparina Street, 4, 117198 Moscow, Russia; 2 Pathology and Clinical Pathology Department, Institute of Human Biology and Pathology, Pirogov Russian National Research Medical University, Ostrovityanova Street, 1, 117279 Moscow, Russia; 3 Pathology Laboratory, Institute of Molecular Pathology and Immunology of University of Porto (IPATIMUP), Rua Júlio Amaral de Carvalho 45, 4200-135 Porto, Portugal; 4 Escola de Medicina e Ciências Biomédicas, Universidade Fernando Pessoa, Praça de 9 de Abril 349, 4249-004 Porto, Portugal; 5 Ivannikov Institute for System Programming of the Russian Academy of Science, Research Center for Trusted Artificial Intelligence, 109004 Moscow, Russia; 6 Department of Pathology, Hospital U Arnau de Vilanova & University of Lleida, Institut de Recerca Biomèdica de Lleida, 28029 Barcelona, Spain; 7 Pathology Department, Medical Faculty of University of Porto, Alameda Prof. Hernâni Monteiro, 4200-319 Porto, Portugal

**Keywords:** computational pathology, gynecological cancer, artificial intelligence, pathology diagnosis

## Abstract

**Background/Objectives**: Accurate diagnosis, prognosis, and prediction of treatment response are essential in managing gynecologic cancers and maintaining patient quality of life. Computational pathology, powered by artificial intelligence (AI), offers a transformative opportunity for objective histopathological assessment. This review provides a comprehensive, user-oriented overview of existing AI tools for the characterization of gynecological cancers, critically evaluating their clinical applicability and identifying key challenges for future development. **Methods**: A systematic literature search was conducted in PubMed and Web of Science for studies published up to 2025. The search focused on AI tools developed for the diagnosis, prognosis, or treatment prediction of gynecologic cancers based on histopathological images. After applying selection criteria, 36 studies were included for in-depth analysis, covering ovarian, uterine, cervical, and other gynecological cancers. Studies on cytopathology and pure tumor detection were excluded. **Results**: Our analysis identified AI tools addressing critical clinical tasks, including histopathologic subtyping, grading, staging, molecular subtyping, and prediction of therapy response (e.g., to platinum-based chemotherapy or PARP inhibitors). The performance of these tools varied significantly. While some demonstrated high accuracy and promising results in internal validation, many were limited by a lack of external validation, potential biases from training data, and performance that is not yet sufficient for routine clinical use. Direct comparison between studies was often hindered by the use of non-standardized evaluation metrics and evolving disease classifications over the past decade. **Conclusions**: AI tools for gynecologic cancers represent a promising field with the potential to significantly support pathological practice. However, their current development is heterogeneous, and many tools lack the robustness and validation required for clinical integration. There is a pressing need to invest in the creation of clinically driven, interpretable, and accurate AI tools that are rigorously validated on large, multicenter cohorts. Future efforts should focus on standardizing evaluation metrics and addressing unmet diagnostic needs, such as the molecular subtyping of rare tumors, to ensure these technologies can reliably benefit patient care.

## 1. Introduction

Worldwide, millions of women of all ages are affected by gynecological cancer, which often leads to significant impairments in general health, quality of life, and, in many cases, early death. Over the recent decades, the classification of gynecological cancers, along with their respective treatment strategies, follow-up protocols, and prognosis for the vast majority of patients, has evolved significantly [1,2,3]. The incidence and mortality of cervical cancer have fallen considerably since human papillomavirus (HPV) was identified as its primary cause and screening and vaccination were introduced [4]. Currently, the greatest focus is on other prevalent cancer types, such as endometrial and ovarian cancers [5]. Despite the advancements in targeted treatments and innovative surgical strategies (including robotic surgery), the mortality rate for endometrial cancer has increased over the past 20 years and has plateaued (≥60%) for ovarian cancer [6].

Today, histopathological diagnostics remain the standard for the classification and therapeutic guidance of gynecological cancers [7]. The integration of computational pathology, supported by artificial intelligence (AI) tools, has the potential to enhance histopathological diagnostic accuracy and provide new prognostic and predictive insights [8,9,10]. Successful AI tools have already been developed for various cancer types (e.g., prostate, breast, and colon cancer) [11,12,13,14]. By utilizing such AI tools, pathologists can have an adjunct to tumor identification, histotyping, staging, and grading, as well as the evaluation of immunohistochemical (IHC) markers and the prediction of IHC results, molecular subtypes, prognosis, and sensitivity to chemotherapy/targeted therapy [15,16,17,18,19].

Over the past three decades, digital images have become increasingly common in medical practice. A major breakthrough in this field occurred in the 1990s with the advent of whole-slide images (WSIs), produced by scanning entire tissue sections on slides rather than focusing on specific regions of interest. Pathologists at different sites could then view these WSIs on a computer monitor, enhancing remote collaboration [20,21,22]. WSIs offer optimized navigation and precision measurement tools, facilitate the exchange of cases between pathologists, and allow the use of computational pathology tools [20,21,22,23,24]. The integration of computational pathology tools, such as those based on AI, into the digital workflow may affect the diagnostic accuracy and efficiency [11]. Moreover, AI outputs would become part of the pathologist’s portfolio, similar to immunohistochemistry, and the acceptance of their results, at this stage, should rely solely on the pathologists. Bringing AI results that are discordant with the pathologist’s observations to the Multidisciplinary Team (MDT) is still at an early stage, as the performance of algorithms still encompasses many errors and is limited to certain organs and pathologies. The discussion of prediction results provided by AI software should definitely take place in MDTs, but, for now, their use is not recommended [25]. In addition, we should mention that, while no FDA-approved digital pathology algorithms yet exist for gynecological diagnostics, future needs for precise molecular subtyping in endometrial and ovarian cancers will require such tools for clinical trial recruitment. Predicting genetic abnormalities directly from H&E slides would further streamline patient identification. Successful implementation will require standardized image acquisition, clear guidelines, and regulatory collaboration [26,27].

AI technologies are rapidly evolving, and the algorithms first based on deep learning’s simple convolutional neural networks (CNNs) are now based more on complex transformer-based algorithms [28]. In addition, taking into consideration all the new evidence on how the pre-scanning operation may influence the AI performance, detailed specifications for use should be a constant in every computational pathology product so that bias may be minimized [29].

This review outlines the current computational-pathology-based approaches to the histological diagnosis, prognosis, and treatment of gynecologic cancers, as well as the challenges and future directions in the field. Cytological diagnosis remains outside the scope of this review, due to the fact that digital cytology is currently an independent field, with commercial solutions and scientific developments so numerous and extensive that they require separate consideration. Furthermore, several comprehensive review publications are already dedicated to this topic [30,31,32,33].

Another limitation of this study is that, over the past 10 years of active development in digital pathology—during which most of the AI tools examined were developed—the approaches to female reproductive tract tumor classification have changed. This is particularly true for ovarian tumors, making it difficult to fully compare AI tools developed using previous classification systems with those designed according to current classification groups. In addition, as a limitation of the study, we recognize that there is an oversimplification of molecular subtyping: although well-known molecular classes (e.g., POLE-mutant and p53-abnormal) are mentioned, epigenetic features and methylation profiles are ignored despite their growing importance. This is because there are no dedicated algorithms to target these less frequent molecular alterations. Moreover, the utility of AI to prioritize variants of unknown significance (VUSs) for secondary review could be proposed as a novel frontier.

## 2. Methodology

A search query was developed in the PubMed and Web of Science databases for literature on this topic published up to and including 2025. The search string was as follows: (Pathology OR Histopathology OR Histopathological OR Whole slide image OR WSI OR Artificial Intelligence OR AI OR Neural Network OR NN or Computational Pathology OR Digital Pathology) AND (Gynecologic Pathology OR Reproductive System Pathology OR Endometrial cancer OR Endometrial carcinoma OR Ovarian cancer OR Ovarian carcinoma OR Fallopian tube cancer OR Fallopian tube carcinoma OR Fallopian tube tumors OR Cervical cancer OR Cervical carcinoma OR Vulvar cancer OR Vulvar carcinoma OR Vaginal cancer OR Vaginal carcinoma OR Uterine cancer OR Uterine carcinoma OR Uterine mesenchymal tumors OR trophoblastic disease OR hydatidiform mole). The selection criteria consisted of accepting only studies that involved the development of AI tools for the diagnosis, prognosis, or prediction of gynecologic cancers. The two databases provided a total of 4360 results, of which 72 were duplicates, and 4252 studies were not included as they did not meet the acceptance criteria.

The remaining 36 studies were analyzed in this review (17 related to ovarian and fallopian tube cancer, 16 to uterine cancer, 2 to cervical cancer, and 1 to lower reproductive tract cancer).

## 3. Computational Pathology Dedicated to Gynecological Cancers

**Figure 1:**
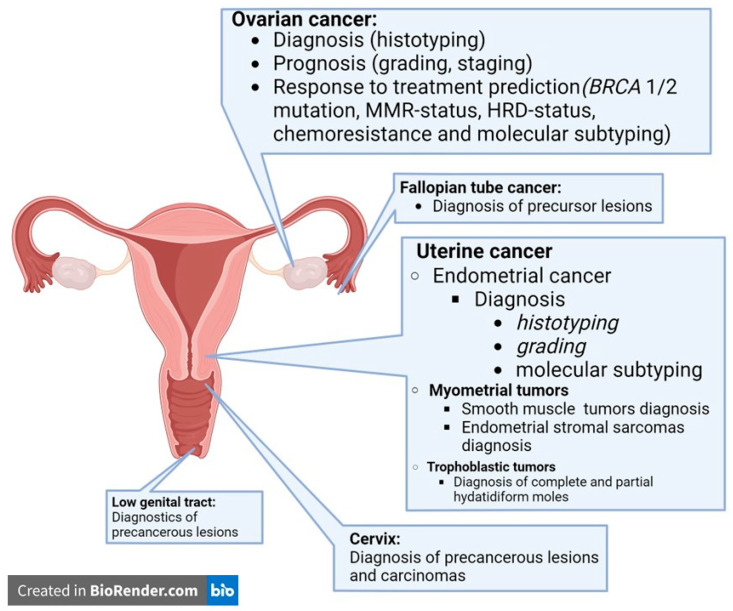
Artificial intelligence tools for characterization of gynecological cancers reported in the literature up to today.

In gynecologic pathology, the applications of AI tools have already been tested for their diagnostic, prognostic, and predictive functions, tailored to the location, molecular subtyping, and target therapy of each cancer. The most frequent AI tools for the characterization of gynecologic cancers are summarized in Figure 1.

## 4. Ovarian Cancer

### 4.1. Diagnosis

There are three main groups of ovarian tumors, classified according to the World Health Organization (WHO) 5th Edition Classification (2020): epithelial, stromal, and germ cell tumors. The most frequent and deadliest are epithelial tumors, which include high-grade serous cancer (HGSC); low-grade serous cancer (LGSC); mucinous, endometrioid, clear cell, and seromucinous cancer; malignant Brenner tumors; and other rare types. Most AI tools for the diagnosis of ovarian cancer are designed to identify the five most common types of epithelial cancer (HGSC, LGSC, and endometrioid, mucinous, and clear cell cancer) (Table 1). Both convolutional neural network (CNN) and support vector machine (SVM) models have been used in the development of these tools. Most of these models use hematoxylin and eosin (H&E)-stained whole-slide images (WSIs) divided into patches, with the most popular sizes being 500 × 500 pixels [34,35] or 256 × 256 pixels [36,37]. However, the final algorithms more frequently operate on WSIs [34,35,38,39] rather than patches [36,37,40]. The metrics used to evaluate the precision of these models vary, which poses challenges for direct comparisons. Nevertheless, all algorithms included in this study achieve an area under the curve (AUC) greater than 0.92 or an accuracy exceeding 90%, although some did not use independent cohorts [34,35,37,40]. With refined training, the inclusion of additional histotypes, and the optimal performance in generalization tests, these AI tools are strong candidates for clinical practice. While some ovarian cancer histotypes have high inter-observer reproducibility, others have a moderate or even low agreement [41,42].

### 4.2. Prognosis

AI-based grading and staging on H&E-stained slides have been proposed to improve the diagnostic accuracy of pathologist evaluations (Table 1). The most relevant grading evaluation for ovarian cancer is the differentiation between HGSC and LGSC, as these tumors are considered different histotypes. Nonetheless, endometrioid and mucinous cancers also require accurate grading. Staging ovarian cancer remains one of the most controversial issues, as it is sometimes difficult to identify the original source of the tumor. Moreover, distinguishing between invasive and non-invasive implants in LGSC can significantly influence the final stage, though the reproducibility of their evaluation is rather low.

The study by Yu et al. was based on The Cancer Genome Atlas (TCGA) cohort, included only serous cancer [43], and focused on histotyping (HGSC and LGSC). The AUC of the AI tool reported in this study was 0.812, which is lower than other models and needs to be improved to be comparable with those achieving AUCs of 0.95–0.972 and also including independent cohorts [38,39]. The staging algorithm proposed by Ghoniem et al. demonstrated a very high accuracy (around 99%) [44]. This study also used the TCGA cohort, in which the majority of tumors were HGSC, whereas pathologists encounter more difficulties with staging LGSC, and endometrioid and mucinous cancers, which could be underrepresented in this study [45,46].

In ovarian cancer, the prediction of the prognosis is crucial for stratification in clinical trials, as most cases are diagnosed at an advanced stage [46,47]. Besides grading and staging, other factors can be used for prognostication, including IHC markers, and various -omics data related to histopathologic image analysis [48,49] (Table 1). The inclusion of different histotypes within the same study may lead to poor results due to the presence of many confounding factors that can affect the final outcome and reduce the accuracy of the prognosis prediction. The study by Poruthoor et al. used three ovarian cancer datasets (genomic, proteomic, and imaging) retrieved from The Cancer Genome Atlas (TCGA) to determine if the prediction of the ovarian cancer grade or patient survival rate can be predicted with AI tools. It was conducted over a decade ago, when CNNs were not as advanced as they are today [50].

A recent study by Yang et al. [51] presents more conclusive results, showing significant statistical differences between two ovarian cancer clusters subdivided according to the Ovarian Cancer Digital Pathology Index (OCDPI). The construction of the OCDPI involves two steps: a histopathological feature extractor and a graph-based deep-learning aggregation module, which integrates embeddings from all patches of the WSI. The use of diverse cohorts (TCGA, the Prostate, Lung, Colorectal, and Ovarian Cancer Screening Trial, and the Harbin Medical University Cancer Hospital), well-organized external validation, and contemporary neural network approaches with a transformer deep-learning architecture has led to much more impressive outcomes [51].

### 4.3. Response to Treatment Prediction

Predicting the treatment response in ovarian cancer is crucial, given the low overall survival and progression-free survival rates of patients with this disease. The accurate identification of chemotherapy-resistant tumors, the evaluation of the potential efficacy of immunotherapy, and enrolling patients in suitable clinical trials with targeted or experimental agents are essential. In ovarian cancer patients, the mutations in BRCA1/2 and mismatch repair (MMR) genes, as well as homologous recombination deficiency (HRD) status and platinum resistance of the tumor, should be evaluated to predict the treatment response. It is well-known that patients with BRCA1/2 mutations can benefit from poly(ADP-ribose) polymerase (PARP) inhibitors [52,53]. Although only a small proportion of ovarian cancers are MMR-deficient (about 16%), these patients may benefit from checkpoint inhibition therapy. The HRD status can predict the response to platinum-based therapy and PARP inhibitors [54,55,56]. Several clinical studies have focused on accurately predicting the response to platinum-based therapy in patients with HGSC, as this is the first-line treatment for the majority of ovarian cancer patients [57]. Therefore, screening for these genetic disorders at the time of diagnosis using AI tools based on H&E-stained slides could be practical for the initial stratification and for further molecular genetic studies to confirm the specific tumor biology (Table 2).

Despite the long history of BRCA1/2 testing and the definition of reliable morphological characteristics [58], the currently developed AI tools for predicting their mutational status are not yet accurate enough for routine practice. Only the study by Zeng et al. demonstrated a high accuracy (0.912) [17], differing from others by using a random forest model and a patient-based predictive approach, while other studies used more traditional models and WSI-based predictions, despite recruiting more patients [18,59,60]. Zeng et al.’s study was not solely dedicated to BRCA1/2 mutation tumors but also included a prediction of the MMR status; however, the study focused only on HGSC and did not provide information on the number of MMR-deficient (dMMR) tumors. An MSI-high status is rare in HGSC and LGSC, and even endometrioid ovarian cancer shows dMMR in fewer than 20% of cases [19]. Nonetheless, the AUC results for the prediction of dMMR were also very high in this study [17], warranting further verification by others. This study is the only one assessing the MSI status in ovarian cancer using AI tools. The pan-cancer investigation by Arslan et al. on the deep-learning-based prediction of multi-omic biomarkers included ovarian cancer but focused on MMR gene mutations in gastrointestinal cancer [61]. Other cross-cancer surveys on the MSI status in solid tumors have only addressed endometrial cancer among gynecological malignancies [62,63].

The HRD status is predominantly associated with BRCA1/2 mutations in ovarian cancer, though some studies consider this parameter separately for the development of AI tools. The pan-cancer study by Loeffler et al. included ovarian cancer cohorts, achieving a reasonable accuracy only for endometrial cancer among gynecological malignancies [64]. Another study by Frenel et al. on HRD status prediction based on whole-slide imaging focused solely on ovarian cancer, achieving an AUC of 0.74 in the proprietary “Discovery cohort” and 0.67 in the TCGA cohort [65]. However, this study is not fully published yet (currently only an abstract is available), making it difficult to assess the strengths and weaknesses of this algorithm. At the same time, the most recent results related to HRD status prediction showed a 72% accuracy for internal cohorts and 57% for external cohorts in the study by Marmé et al. [66]; Bregstrom et al. achieved an AUC of 0.81 for the internal cohort [67], and Zhang et al. reported an AUC of 0.769 [68].

Several AI tools have been developed to predict the response to chemotherapy by combining clinical, molecular, and genetic data. Most proposed algorithms related to platinum-based therapy effectiveness have demonstrated a high accuracy (above 90%) and/or AUC (above 0.95) [43,69,70]. Yu et al.’s studies aimed not only to predict the chemotherapy response but also to distinguish the molecular subtypes of high-grade ovarian cancer [43]. Gilley et al. demonstrated how to predict the effectiveness of bevacizumab in ovarian cancer treatment using a pathomics biomarker; however, the final results were not particularly impactful (AUC 0.82–0.83) [71]. Meanwhile, the prediction of PARP-inhibitors’ efficacy was less successful: Marmé et al. demonstrated an only 72% accuracy for internal cohorts and 57% for external cohorts [66].

Molecular subtyping is critical for both the prognosis and treatment stratification of patients with HGSC. Previous approaches have been developed to distinguish these subtypes (immunoreactive, mesenchymal, differentiated, and proliferative) based on molecular and genetic data, morphological features, and IHC characteristics [15,72]. However, the results of this study were moderate, with a Spearman correlation with true molecular subtypes ranging from 0.111 to 0.576 [43]. The development of dedicated AI tools for subtyping LGSC could improve the outcomes of targeted treatment with mitogen-activated protein kinase (MEK) inhibitors and enhance prognostication.

**Table 1:** AI tools for ovarian cancer diagnosis, prognosis, and response to treatment.

	Patients/ Original Images (n)	Original Image Type	Image for AI Training Size (Pixels)	Features to Be Assessed/Final Model	AI Tool Input	AI Tool Output	Internal Results Metrics	Internal Results
**Histotyping**								
BenTaieb et al., 2016 [34]	80/80	WSI	500 × 500	Color, texture, cellular morphology, cytology/SVM	WSI	5 classes *	Accuracy	95%
BenTaieb et al., 2017 [35]	133/133	WSI	500 × 500	CNN features novel K-means/SVM	WSI	5 classes *	Accuracy	90%
Levine et al., 2020 [36]	406/406	WSI	256 × 256	CNN VGG19	patch	5 classes *	Accuracy	70.87%
							AUC	0.92
Kasture et al., 2020 [37]	≤500/500	patch	N/D	CNN novel KK Net	patch	5 classes *	Accuracy	91%
							AUC	0.95
Boschman et al., 2022 [38]	160/308	WSI	256 × 256	CNN ResNet 18	WSI	5 classes *	AUC	0.97
Farahani et al., 2022 [39]	485/948	WSI	512 × 512	CNN VGG19	WSI	5 classes *	AUC	0.95
Idlahcen et al., 2025 [40]	500/500	WSI	224 × 224	Autoencoder + CNN (DenseNet-201)	patch	5 classes *	Accuracy	94.88%
**Staging and grading**								
Yu et al., 2020 [43]	80/80	WSI	N/D	CNN VGG16	WSI	2 grades (low/moderate and high)	AUC	0.812
Ghoniem et al., 2021 [44]	160/308	WSI	256 × 256	CNN altered VGG16	WSI	5 FIGO stages (I–IV) and N/D	Accuracy	98.87%
**Prognosis**								
Poruthoor et al., 2013 [50]	382/≤382	WSI	512 × 512	CNN features/novel SVM	WSI	2 classes of survival rate (<5 years/ ≥5 years	Accuracy	55%
Yang et al., 2024 [51]	874/1826	WSI	224 × 224	Transformer network/graph deep-learning analysis	WSI	2 classes of survival rate (high and low OCDPI)	Comparison of survival rate	<0.001
***BRCA1*/*2* mutation status**								
Zeng et al., 2021 [17]	229/≥229	WSI	256 × 256	CNN features VGG19/ random forest	WSI	2 classes of BRCA mutations (BRCA_mut_ and BRCA_wt_)	AUC	0.912
Nero et al., 2022 [59]	664/664	N/D	256 × 256	CNN Features ResNet50/ CNN (CLAM)	WSI	2 classes of BRCA mutations (BRCA_mut_ and BRCA_wt_)	AUC	0.59
Ho et al., 2023 [60]	609/609	WSI	224 × 224	CNN features novel KK Net/CNNResNet 182	WSI	2 classes of BRCA mutations (BRCA_mut_ and BRCA_wt_)	AUC	0.43
Borgade et al., 2023 [18]	867/867	WSI	512 × 512	PyTorch 3.7, Deepflash2 U-Net, DeepLabv3, UNet++, LinkNet, ResNet, Inception, EfficientNet, ResNeSt	WSI	2 classes of BRCA mutations (BRCA_mut_ and BRCA_wt_)	AUC	0.681
**MMR mutation status**								
Zeng et al., 2021 [17]	229/≥229	WSI	1000 × 1000	texture, cellular, and nuclear morphology/random forest	WSI	3 classes of MMR status (MSI high/ MSI stable/N/A	AUC dMMR	0.919
							AUC pMMR	0.924
**HRD status**								
Loeffler et al., 2023 [64]	520/520	WSI	224 × 224	ResNet50 (pretrained) + attMIL	WSI	2 classes: HRD-high/low and HRD prediction score	AUROC	0.61
Frenel et al., 2024 [65]	244/≥244	WSI	N/D	Fusion-like DNN	WSI	2 classes: HRD+/− and HRD prediction score	AUC (internal)	0.74
							AUC (external)	0.67
Marmé et al., 2025 [66]	669/675	WSI	224 × 224	ResNet18&Transformer	WSI	2 classes HRD status positive/negative and HRD prediction score	AUROC (internal)	72%
							AUROC (external)	57%
Bergstrom et al., 2024 [67]	600/≥1356	WSI	256 × 256	Multiresolution MIL-ResNet18	WSI	classes: HRD+/− and HRD prediction score	AUC (internal)	0.81
							AUC (external)	N/D
Zhang et al., 2025 [68]	205/205	WSI	512 × 512	UNet++ and Hover-Net	patch	HRD status (deficient/proficient) at WSI level	AUC	0.769
							F1-score	0.762
**Chemotherapy response 2prediction**								
Wang et al., 2023 [65]	<180/180	WSI	512 × 512	CNN features novel/SVM	TMA	2 classes of CT efficacy (effective/ invalid)	Accuracy	90%
Wang et al., 2022 [69]	78/288	WSI	256 × 256	CNN (Inception V3)	WSI	2 classes of CT efficacy (effective/ invalid)	AUC	0.99
Yu et al., 2020 [43]	570 ≤ 1358	WSI	N/D	CNN features VGG16	WSI	2 classes of relapse (early/late relapse)	AUC	0.95
							Accuracy	91%
Gilley et al., 2024 [71]	78/288	WSI	1000 × 1000	SVM	patch	2 classes of relapse (responders/nonresponders)	AUC (linear SVM)	0.83
							AUC (Gaussian SVM	0.82

* 5 classes include HGSC, LGSC, and endometrioid, mucinous, and clear cell cancer. WSI—whole-slide image; CNN—convolution neural network; SVM—support vector machine; AUC—area under the curve; N/D—no data; OCDPI—Ovarian Cancer Digital Pathology Index; CT—chemotherapy; MMR—mismatch repair; MSI—microsatellite instability; attMIL—Attention-based Multiple Instance Learning.

**Table 2:** AI tools for endometrial cancer histotyping, grading, and molecular subtyping.

	Patients/ Original Images (n)	Original Image Type	Image for AI Training Size (Pixels)	Features to Be Assessed/ Final Model	AI Tool Input	AI Tool Output	Internal Results Metrics	Internal Results
**Histotyping**								
Hong et al., 2021 [73]	456/ 20,000	WSI	299 × 299	Inception Resnet-based/CNN	WSI, patch	2 histotypes (endometrioid/ serous)	AUROC patient	0.969
							AUROC patch	0.870
Song et al., 2022 [74]	109	WSI	360 × 360	Inception-v3	WSI	2 histotypes (endometrioid/ serous)	AUROC	0.944
**Grading**								
Goyal et al., 2024 [75]	929/ N/D	WSI	N/D	EndoNet	WSI	2 grades: low/high	F1-score	0.91
							AUC	0.9
**Immunohistochemical markers automatic assessment**								
Kildal et al., 2024 [76]	1228/ 2456	WSI	800 × 800	YOLOv5 for nuclear model	patch	2 classes of nuclei as (positive/negative); fraction of positive tumor cells	CCR (PMS2)	95.3%
							CCR (MSH6)	90.0%
							CCR (MSI, combined PMS2 and MSH6)	90.7%
Ji et al., 2024 [77]	57/114	WSI	256 × 256	U-Net and DenseNet-121	patch	Digitally generated H-DAB IHC-stained images	Rintraslide validation:	0.98
							Rcross-case validation	0.66
**Molecular subtypes**								
Hong, 2021 [73]	456/ 20,000	WSI *	299 × 299	Inception Resnet-based CNN	WSI, patch	4 molecular subtypes	AUROC patient CNV-L	0.889
							AUROC patch CNV-L	0.710
							AUROC patient CNV-H	0.873
							AUROC patch CNV-H	0.713
							AUROC patient MSI-H	0.827
							AUROC patch MSI-H	0.638
Fremond, 2023 [78]	2028/ 1,170,931	WSI	224 × 224	ResNet 50, MoCo-v2	WSI	POLE	AUROC	0.849
						dMMR	AUROC	0.844
						NSMP	AUROC	0.883
						p53 abn	AUROC	0.928
Goyal, 2024 [79]	2072/ 3,702,447	WSI	224 × 224	HECTOR	WSI	2: low/high grade	F1-score	0.91
							AUC	0.95
**MMR status**								
Zhang, 2018 [75]	N/A	N/A	1000 × 1000	Inception-V3	WSI	2 (MSI, MSS)	Accuracy	84.2%
Kather, 2019 [80]	81/94	WSI	N/D	ResNet18	Patch	2 (MSI, MSS)	AUC	0.75
Wang, 2020 [81]	N/A	N/A	512 × 512	ResNet18	WSI	2 (MSI, MSS)	AUC	0.73
Zhang, 2023 [75]	95/ 22,044	WSI	256 × 256	ResNet34 VGG16	Patch	2 (MSI, MSS)	AUC	0799
							F1-score	0786
Wang, 2024 [82]	344/ N/A	WSI	512× 512	Inception-V3	WSI	2 (MSI, MSS)	AUC	87%
							F1-score	84%
Arslan, 2024 [61]	61/ 12,093 (totally)	WSI	256 × 256	ResNet34	WSI	2 (MSI, MSS)	AUC	0.771
Whangbo et al., 2024 [83]	325/1168	WSI	N/D	EfficientNetB2	patch	2 (MSI, MSS) on WSI level	AUC	0.821
							Accuracy	0.778
Umemoto et al., 2024 [84]	114	WSI	512 × 512	ResNet50	patch	2 (MSI, MSS) on WSI level	AUC	0.91
							Accuracy	0.80
Liu et al., 2025 [85]	1027/1678	WSI	224 × 224	ResNet18 and EfficientNet	patch	2 (MSI, MSS) on WSI level via patch-level probability averaging.	AUC (internal)	0.897
							AUC (external)	0.790–0.863

* H/E and IHC WSI were used; N/A—not available; H/E—hematoxylin and eosin; WSI—whole-slide image; CNN—convolution neural network; POLE—Polymerase ɛ; dMMR—Deficient DNA Mismatch Repair; NSMP—no specific molecular profile; AUROC—area under the receiver operating characteristic; AUC—area under the curve; CCR—Correct Classification Rate.

## 5. Fallopian Tube Cancer

The most common fallopian tube malignancy is high-grade serous carcinoma; however, tumors confined to the fallopian tube are rare, and tubal HGSC is predominantly diagnosed at an advanced stage after spreading to the ovaries and peritoneum [86,87]. Consequently, some authors recommend the term “pelvic HGSC” because it is often challenging to determine the original source of such cancer [86,88]. A putative precursor of tubal HGSC, known as serous tubal intraepithelial carcinoma (STIC), has been described and is located in the endosalpinx of the fallopian tube, predominantly in the fimbrial part [89]. Detecting STIC or early-stage tumors in fallopian tubes removed by prophylactic or opportunistic salpingectomy can prevent the development of HGSC [90,91]. A two-step procedure, involving morphological and IHC determination with an assessment of p53 and Ki-67 expression, has been proposed for accurate and reproducible STIC diagnosis [92]. Bogaerts et al. explored this procedure using a neural-network-based model with U-net and ResNet50. The final algorithm was developed for the WSI-based verification of two classes (serous tubal intraepithelial lesion (STIL) and STIC), but the F1-score was rather low (0.35) [93]. This is likely only a pilot model, but the unsatisfactory result could be partly explained by the very small size of the lesions (<30 cells) and the difficulties in slide mapping.

## 6. Uterine Cancer

### 6.1. Endometrial Cancer

Endometrial cancer has undergone changes in its WHO classification [7], molecular subtyping, and risk stratification [3]. A new International Federation of Gynecology and Obstetrics (FIGO) staging system (2023) has been published [94], and new treatment strategies have been proposed, including the prominent role of immunotherapy [95], providing several opportunities for the application of AI tools [96]. Currently, most AI tools focus on detecting only two histotypes (endometrioid and serous), while other histotypes (such as clear cell cancer or carcinosarcoma) are overlooked [73,74]. Additionally, there is a lack of AI tools for detecting the polymerase epsilon (POLE)-mutant molecular subtype, even though these patients could benefit from de-escalated therapy due to the extremely benign nature of this subtype [73,78].

AI tools for histotyping, grading, and staging endometrial cancer are summarized in Table 2. All these algorithms have demonstrated a high predictive value, although the histotyping models only considered the two most common cancer types (endometrioid and serous), which may lead to the misinterpretation of rarer tumors (e.g., clear cell cancer, carcinosarcomas, and mesonephric cancer). There is limited research on predictive algorithms for grading, but Volinsky-Fremond et al. included this evaluation in their enriched cohort, which was also used for predicting the MSI status, showing a cumulative AUC of 0.844 with the Histopathology-based Endometrial Cancer Tailored Outcome Risk (HECTOR) model [97]. Some models have been developed to evaluate the endometrial immunophenotype, as this is an important part for diagnosis and prognosis.

These algorithms were constructed either as a digital IHC algorithm (for digital Ki-67 expression, as demonstrated by Ji et al. [77]) or for the automatic assessment of physically stained slides (for PMS2/MSH6 expression evaluation, as described by Kildal et al. [76]). Both algorithms demonstrated a high internal accuracy (>90%). Nevertheless, the digital IHC algorithm still requires improvement for cross-case validation, as the proposed model showed a Pearson correlation of only 0.66 between digital and physical Ki-67 labelling indices.

Since molecular subtypes were proposed in 2013 [98] and surrogate markers have been developed to distinguish between these subtypes in pathological practice [99], several AI tools have been proposed to evaluate them. Among the molecular subtyping models, the algorithm by Fremond et al. [78] is the most impressive, with a high accuracy for all subtypes, developed using data from more than 2000 patients and over 1 million images. Other models for predicting the MSI status showed reasonably good, but not excellent, accuracy/AUC, though none examined such a large number of patients [61,75,80,81,83,84,100]. The AI tool developed by Fremond et al. could benefit from further training with an independent cohort to achieve high-level performance for clinical use. In pan-cancer studies, endometrial cancers have also been included in prediction models. For instance, Arslan et al. reported an AUC of 0.771 for endometrial cancers, higher than the average AUC of 0.653 [61]. Another pan-cancer study including endometrial cancer cohorts did not analyze the prediction of the MSI status for these tumors, focusing instead on the prediction of PTEN, TP53, and APC mutations dependent of the MSI status [62]. The most recent investigation by Liu et al. [85] demonstrated impactful results, analyzing more than 1000 patients and achieving an AUC of approximately 0.9. In addition, their algorithm showed a high AUC for external cohorts (0.790–0.863), which have not been reported previously.

### 6.2. Uterine Mesenchymal Tumors

#### 6.2.1. Uterine Smooth Muscle Tumors

The morphological criteria—nuclear atypia, necrosis, and mitotic figures—remain the most important features for diagnosing uterine smooth muscle tumors [7,101,102]. Although the classification of atypia is not easily addressed by AI tools due to challenges in establishing the ground truth, the AI-based evaluation of mitotic activity has been widely explored in other tumor models [103,104,105]. In uterine mesenchymal tumors, simple machine-learning-based AI tools have been developed to assess the mitotic index and generally achieve a high predictive value (over 90%).

It has also been demonstrated that uterine leiomyosarcoma can be subdivided into molecular subtypes with different prognoses and potentially different therapeutic targets [106,107,108]. However, we could not find AI tools specifically designed to detect such molecular subtypes.

#### 6.2.2. Uterine Stromal Sarcoma

Some AI tools have been proposed to distinguish uterine stromal sarcomas from other tumors and tumor-like conditions. Yang et al. reported a comparison of different models to differentiate low-grade stromal sarcomas (LGSSs) from leiomyomas [108]. The authors concluded that the neural network PCA (Principal Component Analysis) with an SVM is the most effective of the basic techniques for training their model, achieving a final test accuracy of 0.8535 for LGSS. These technical comparisons are useful for designing the experiments; nevertheless, the overall accuracy of AI tools depends not only on the neural network design but also on the characteristics of the training sets. In the study by Yang et al., there was no information on the size or origin of the cohort, so further research is needed to substantiate this result.

According to the WHO 2020 classification [7], endometrial stromal sarcoma includes many entities with different molecular subtypes that cannot be distinguished based on morphological features alone. This is an area that could benefit from a decision support AI tool.

### 6.3. Trophoblastic Tumors

Trophoblastic tumors are among the most difficult categories to diagnose, as there are few reliable morphological and IHC features to support the differential diagnosis. The fragility of the morphological criteria makes this cancer type less attractive for developing AI tools, since the ground truth is difficult to establish. One of the differential diagnostic challenges in trophoblastic tumors is the correct labelling of hydatidiform moles, as this can significantly affect the follow-up strategy. We found only one AI tool for the diagnosis of trophoblastic tumors. Palee et al. proposed a model for the differential diagnosis of complete and partial hydatidiform moles [109], based on a neural network classifier and machine-learning-based classifiers, with an accuracy of 81.2%, trained on more than 900 images captured from WSI. The accuracy achieved by the Palee et al. model is quite good; however, the differential diagnosis between complete and partial hydatidiform moles is not a significant problem for pathologists, as the p57 IHC expression is an accurate marker that is differentially expressed in these two conditions [110]. Currently, the most important problem in the pathological evaluation of villi is the differentiation between the hydropic abortus (non-hydatidiform mole) and partial hydatidiform mole, as this is not possible without a genetic analysis of short tandem repeats (STRs) from the paraffin-embedded material [111,112]. Although this method has been developed in detail and has been proven to be excellent, many pathology and genetics departments are unable to perform this analysis regularly. An initial screening with an AI tool would provide an effective triage approach for the verification of partial hydatidiform moles.

## 7. Lower Genital Tract

### 7.1. Cervical Cancer

There are not many AI tools for histological cervical cancer diagnosis, partly because the incidence of cervical cancer has decreased dramatically in recent decades due to the introduction of screening and vaccination programs [1,4]. Screening options expanded by digital cytology are now enhanced by dedicated AI tools [30,113,114,115].

The main drawback of AI tools for the histopathological diagnosis of cervical cancer is the patch-based analysis used in some models, which prevents the adoption into routine practice and requires further improvement. Nevertheless, rather accurate AI tools were proposed by Habtemariam et al. [114] and Li et al. [116]. The AI tool developed by Habtemariam et al. was trained on 915 patients (WSI) and demonstrated a 94.5% accuracy for four classes (normal, precancerous (squamous intraepithelial lesions), adenocarcinoma, and squamous cell carcinoma (SCC)) [114]. Li et al. developed an AI tool for distinguishing between only two classes (adenocarcinoma and SCC) with an AUC of 0.966 [116]. Since the 2020 WHO classification focuses on the distinction between HPV-associated and HPV-independent cervical cancers [7], the HPV status should instead be the fundamental feature to target in future AI training. The same applies to the key role of p16, which is a single marker recommended by the Lower Anogenital Squamous Terminology Project for a high-grade squamous lesion diagnosis to improve the sensitivity and specificity of the histopathological diagnosis [117]. An AI-based quantification and spatial assessment of p16 could be very helpful for pathologists, as it is sometimes difficult to distinguish between patchy and block-type p16 staining. An et al. demonstrated an AI tool that can accurately identify HSIL among p16-positive areas with a high accuracy, sensitivity, and specificity (0.845, 0.922, and 0.829, respectively) [118].

### 7.2. Vulvar and Vaginal Cancers

Precancerous lesions of the vulva and vagina are even more difficult to diagnose than cervical lesions, as there is still no uniform classification for HPV-negative p53 wild-type lesions [3]. These lesions have a very similar phenotype, and IHC markers such as p16, p53, and CK17 cannot help to differentiate them [119,120]. HPV-dependent lesions (vulvar intraepithelial neoplasia of the usual type, uVIN) and HPV-independent p53 mutant lesions (differentiated VIN, dVIN) cannot be differentiated in more than 20% of cases based on morphological features alone [121,122]. Currently, the already developed AI tools can only contribute to the distinction between VINI/II/III. Choschzick et al. presented an AI tool for Ki-67 expression assessment in precancerous vulvar lesions. However, the reproducibility between pathologists and the AI tool was rather low, a result that could be partially explained by the mild atypia and marked keratinization in VINs, which is difficult to assess [123].

In summarizing the presented algorithms across different tumor localizations, the key strengths and limitations of this review must be highlighted. This review provides a comprehensive and critical synthesis of a substantial body of literature (125 references) on AI in gynecological pathology. It maintains a clear, clinically oriented focus on the diagnostic, prognostic, and predictive applications relevant to pathologists. The work not only describes existing tools but also critically evaluates their methodologies, identifying common pitfalls like the lack of external validation. It concludes with practical recommendations for future standardization and the safe integration of AI into clinical workflows. Nevertheless, the scope is intentionally limited to histopathology, excluding the vast field of digital cytology. The direct comparison of AI tools is challenging due to the evolving tumor classifications over the past decade, rendering some models outdated. The analysis focuses on common molecular classes, omitting emerging aspects like epigenetics due to a lack of dedicated algorithms. Furthermore, the review does not address the critical impact of pre-analytical variables on AI classifier robustness.

## 8. Conclusions

In general, AI-based-tools for assessing gynecologic cancers should be considered a promising area of computational pathology, already targeting clinically relevant diagnostic, prognostic, and predictive goals. In this review, many accurate models with potential application in clinical routine are identified as supportive tools that remain largely dependent on the initial diagnostic framing by the pathologist (narrow AI). Some studies lack information on their training sets, such as the diseases included, the number of patients, the number of samples or images used, whether patients or images were excluded, and the methods by which tissue findings were reported. Many studies do not report the external validation results and lack cross-validation. In addition, some studies used inappropriate datasets, including frozen tissue sections, datasets without sufficient classes or histotypes, or unbalanced frequencies of cancers that do not reflect reality, and/or do not include challenging or rare cancer types. These observations highlight the clear need for a standardized algorithm specification for each AI tool.

The works included in this review reported different metrics to evaluate the effectiveness of the proposed AI tools (accuracy, AUC, F1-score, kappa, etc.), which makes it difficult to compare the models. Establishing standardized metrics would make the evaluation and comparison clearer for users. Some researchers also chose technology that is now outdated for the development of their models, so their results could be partially improved by using updated technology. However, the most important problem in computational gynecologic pathology is the lack of investment by researchers in issues that are essential for pathologists in routine practice. Consequently, AI tools for clinically relevant subtyping (e.g., the molecular subtyping of LGSC, leiomyosarcomas, and high-grade stromal sarcomas of the uterus; hydatidiform moles and vulvar precancerous lesions) represent areas where accurate, explainable, and generalizable AI tools would have a positive impact on clinical practice for the benefit of patients. At the same time, the already developed algorithms that have demonstrated convincing results in tasks such as the classification of histological subtypes, tumor grading, prognosis determination, and potential sensitivity to chemotherapy and targeted therapy must be validated and tested on larger patient cohorts using scanners from different manufacturers and slides prepared in various laboratories in order to be fully standardized before being implemented into clinical practice. Finally, future research should not only examine patients’ psychological responses and clinicians’ readiness to use AI but also adopt structured evaluation frameworks to ensure AI systems are integrated into oncology workflows safely, transparently, and equitably. As proposed by Erul et al. [124], implementing a quality assurance framework with standardized reporting, explainability mechanisms, and external validation can enhance trust among both patients and physicians. Such frameworks are essential in low-resource settings, where deploying unvalidated or non-transparent AI tools could exacerbate existing healthcare disparities.

## Data Availability

No data was generated.

## Abbreviations

The following abbreviations are used in this manuscript:

AI	Artificial intelligence
AUC	Area under the curve
CNN	Convolutional neural network
CTransPat	Transformer-based unsupervised contrastive learning for histopathological image classification
dMMR	MMR deficient mismatch repair
dVIN	Differentiated VIN
FDA	Food and Drug Administration
FIGO	The International Federation of Gynecology and Obstetrics
H&E	Hematoxylin and eosin
HECTOR	Histopathology-based Endometrial Cancer Tailored Outcome Risk
HGSC	High-grade serous cancer
HPV	Human papillomavirus
IHC	Immunohistochemical
LGSC	Low-grade serous cancer
LGSS	Low-grade stromal sarcoma
MDT	Multidisciplinary Team
MEK	Mitogen-activated protein kinase
ML	Machine learning
PAIP	Pathology Artificial Intelligence Platform
PARP	Poly(ADP-ribose) polymerase
PCA	Principal component analysis
POLE	Polymerase epsilon
RetCCL	Retrieval with Clustering-guided Contrastive Learning
SCC	Squamous cell carcinoma
STIC	Inhibitors serous tubal intraepithelial carcinoma
STIL	Serous tubal intraepithelial lesion
STRs	Short tandem repeats
SVM	Support vector machine
TCGA	The Cancer Genome Atlas
uVIN	Vulvar intraepithelial neoplasia of usual type
WHO	World Health Organization
WSI	Whole-slide imaging
